# Programmed death‐ligand 1 expression and prognostic significance in bevacizumab treated ovarian cancer patients: Results from the phase IV MITO16A/MaNGO OV‐2 translational study

**DOI:** 10.1002/ctm2.70373

**Published:** 2025-06-22

**Authors:** Francesca Basso‐Valentina, Vincenzo Canzonieri, Rossella De Cecio, Laura Arenare, Domenica Lorusso, Sabrina Chiara Cecere, Eliana Pivetta, Daniela Russo, Annabella Di Mauro, Grazia Artioli, Anna Spina, Carmine De Angelis, Daniela Califano, Saverio Cinieri, Giosuè Scognamiglio, Vanda Salutari, Paolo Chiodini, Francesco Perrone, Sandro Pignata, Gustavo Baldassarre

**Affiliations:** ^1^ Molecular Oncology Unit, Department of Research and Diagnostic Centro di Riferimento Oncologico di Aviano (CRO), IRCCS, National Cancer Institute Aviano Italy; ^2^ Pathology Unit, Department of Research and Diagnostic Centro di Riferimento Oncologico di Aviano (CRO), IRCCS, National Cancer Institute Aviano Italy; ^3^ Department of Medical, Surgical and Health Sciences University of Trieste Trieste Italy; ^4^ Pathology Unit Istituto Nazionale Tumori IRCCS ‐ Fondazione G. Pascale Naples Italy; ^5^ Clinical Trials Unit Istituto Nazionale Tumori IRCCS ‐ Fondazione G. Pascale Naples Italy; ^6^ Department of Life Science and Public Health Catholic University of Sacred Heart Largo Agostino Gemelli and Fondazione Policlinico Universitario A. Gemelli, IRCCS Rome Italy; ^7^ Uro‐Gynecological Medical Oncology Istituto Nazionale Tumori IRCCS ‐ Fondazione G. Pascale Naples Italy; ^8^ Microenvironment Molecular Targets Unit Istituto Nazionale Tumori IRCCS ‐ Fondazione G. Pascale Naples Italy; ^9^ Oncologia Medica ULSS 2 Marca Trevigiana Treviso Italy; ^10^ Department of Medical Oncology School of Medicine and Surgery University of Naples Federico II Naples Italy; ^11^ Medical Oncology Division and Breast Unit Senatore Antonio Perrino Hospital, ASL Brindisi Brindisi Italy; ^12^ Scientific Directorate Istituto Nazionale Tumori IRCCS ‐ Fondazione G. Pascale Naples Italy; ^13^ Department of Mental Health and Public Medicine Section of Statistics Università degli Studi della Campania Luigi Vanvitelli Naples Italy

1

Dear Editor,

We are pleased to present our latest analyses, showing that multiple immunofluorescence (MIF) can be used in large multicenter clinical trials to define the prognostic/predictive value of programmed death‐ligand 1 (PD‐L1) by concomitantly and precisely assessing its expression and spatial distribution in the tumour tissue, outperforming classical immunohistochemistry (IHC).

Immunotherapy with Immune Checkpoint Inhibitors (ICIs) has revolutionized cancer treatment, becoming the primary therapeutic option for several types of tumours.^1^ One of the main ICIs targets is the PD‐1/PD‐L1 axis, and the assessment of PD‐L1 expression by IHC is used as a predictive biomarker of response. Although ICIs have not proven successful for Epithelial Ovarian Cancer (EOC) patients and PD‐L1 expression did not show a predictive value,^2^, ^3^, ^4^ new results of the Keynote‐B96 trial suggest otherwise and support the research for better biomarkers of ICIs activity. Accordingly, while new studies have explored the combination of ICIs with targeted agents in EOC—such as PARP inhibitors and the anti‐angiogenic agent bevacizumab (BEV)—and are supported by growing evidence that BEV can modulate the tumour microenvironment (TME) and enhance ICIs efficacy through synergistic effects, these efforts have largely failed due to the lack of reliable predictive biomarkers to identify patients most likely to benefit.^5^, ^6^ Here, we analyzed by Multiple Immuno‐Fluorescence (MIF) the expression and spatial localization of immune cells and PD‐L1 in 292 samples from patients enrolled in the prospective phase IV MITO16A‐MaNGO OV‐2 clinical trial, which aimed to explore the prognostic role of selected clinical and biological factors in EOC patients treated in first line with standard chemotherapy plus BEV (Table 1 and Figure S1).^7^ The choice of using MIF relies on the possibility of precisely assessing biomarkers’ spatial distribution in the tissue and of defining which cell types express PD‐L1 in the tumour or the surrounding stroma.

**TABLE 1:** Clinical and pathological characteristics of patients in analysis.

	Population enrolled (*n* = 398)		Patients in analysis for IHC (*n* = 100)		Patients in analysis for MIF (*n* = 292)	
	59.1(49.8–66.5)		60.7 (48.8–66.5)		59.2 (49.8–66.5)	
**Median age (IQR)**	***n***	**(%)**	***n***	**(%)**	***n***	**(%)**
**Age category**						
** < 65**	278	(70)	67	(67.0)	202	(69.2)
**≥65**	120	(30)	33	(33.0)	90	(30.8)
**ECOG performance status**						
0	315	(79.2)	87	(87.0)	234	(80.1)
1	69	(17.3)	13	(13.0)	51	(17.5)
2	14	(3.5)	0	(0)	7	(2.4)
**Residual disease**						
None	153	(38.4)	42	(42.0)	115	(39.4)
≤ 1 cm	72	(18.1)	22	(22.0)	60	(20.5)
> 1 cm	120	(30.2)	31	(31.0)	90	(30.8)
Not operated	53	(13.3)	5	(5)	27	(9.2)
**FIGO stage**						
IIIB	36	(9.1)	11	(11.0)	27	(9.2)
IIIC	275	(69.1)	70	(70.0)	207	(70.9)
IV	87	(21.9)	19	(19.0)	58	(19.9)
**Tumor histology**						
High Grade serous	333	(83.7)	82	(82.0)	254	(87.0)
Low Grade serous	13	(3.3)	6	(6.0)	9	(3.1)
Endometrioid	9	(2.3)	3	(3.0)	8	(2.7)
Clear Cell	11	(2.8)	3	(3.0)	10	(3.4)
Mucinous	3	(0.8)	0	(0)	1	(0.3)
Mixed	4	(1.0)	2	(2.0)	2	(0.7)
Other	25	(6.3)	4	(4.0)	8	(2.7)

IHC = Immunohistochemistry.

MIF = Multiple Immuno‐Fluorescence.

IQR = Interquartile Range.

We first compared two different anti‐PD‐L1 antibodies, and assessed their concordance in scoring PD‐L1 on 100 selected samples, using IHC which is the gold standard for PD‐L1 evaluation in the clinic.^8^ Statistical analyses showed high correlation and concordance between two independent pathologists and the two antibodies (Figure 1A–G). The anti‐PD‐L1 E1L3N antibody demonstrated slightly better performance in scoring PD‐L1 positivity and was selected for MIF analyses (Figure 1H and Figure S2). The correlation and the agreement between MIF and IHC using the E1L3N antibody were very high, demonstrating that the evaluation of PD‐L1 by MIF is comparable to classical IHC (Figure 1I‐L) although, as expected, the percentage of positive cells decreased approximately 40‐fold in MIF computer‐assisted count compared to the human evaluation (Supporting Information results). On these bases, we moved to large‐scale MIF analyses and stained 326 samples from 292 patients for the expression of CD8 (tumour infiltrating T cells), CD68 (tumour infiltrating monocyte/macrophages), cytokeratins (tumour cells), and nuclei, along with CD274 (PD‐L1) (Figure 2A). Five color‐stained slides were studied using a multispectral camera and computer‐assisted analyses. Pearson correlation analyses demonstrated a high correlation in PD‐L1 expression between primary and metastatic lesions in 32 patients who donated both samples (*r* = 0.83; *p* < 0.0001). Sixty‐three per cent of the analyzed samples were positive for PD‐L1 expression, and most of the positive samples had PD‐L1‐positive cells both in the tumour and the stroma (Figure 2B). By analyzing the expression and localization of PD‐L1, we observed that the majority of PD‐L1‐positive samples were also positive for CD68 (64%), suggesting that the major source of PD‐L1 in the analyzed EOC samples was due to the presence of cells from the monocyte/macrophage lineage. Among PD‐L1‐positive samples, 6% contained cells that expressed PD‐L1 only on the tumour cells (PD‐L1^+^/CK^+^), while 23% of the samples had cells that were PD‐L1^+^/CD68^+^ only. Finally, for 30% of the samples, we were unable to identify the cell subtype that expressed PD‐L1 (Figure 2C). PD‐L1 negative samples were mostly CD68 positive, further supporting the notion that cells from the monocyte/macrophage lineage were the immune cells predominantly present in EOC samples.^9^

**FIGURE 1:**
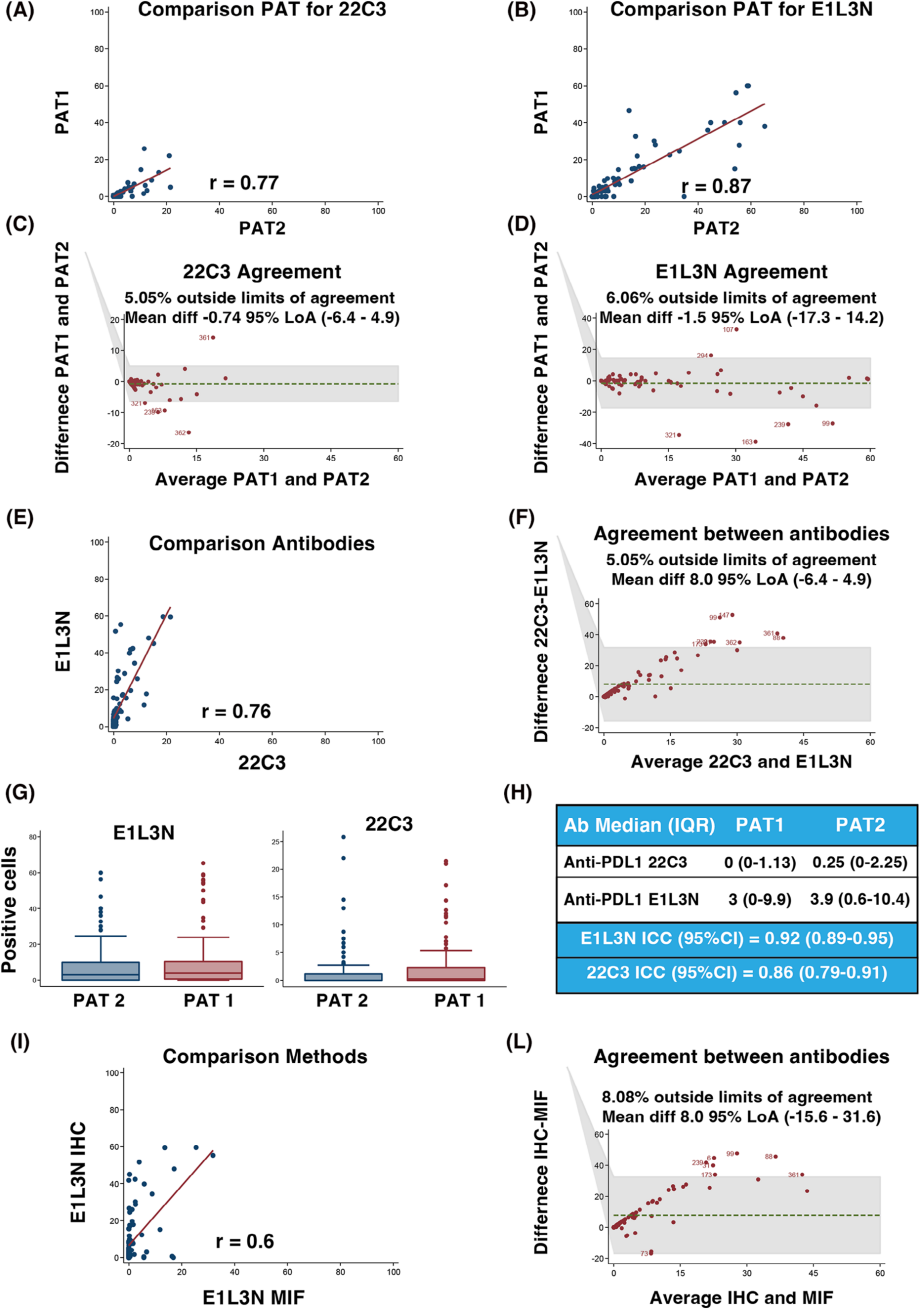
Correlation and agreements between different anti‐PD‐L1 antibodies and staining techniques. A/B Correlation analyses evaluating the scoring of Pathologist 1 (PAT1) and 2 (PAT2) in evaluating the staining of PD‐L1 using the 22C3 (A) or the E1L3N (B) antibodies in immunohistochemistry. C/D Graphs reporting the Bland‐Altman analysis evaluating the Limits of Agreement (LOAs) between PAT1 and PAT2 in evaluating PD‐L1 expression using the 22C3 (C) or the E1L3N (D) antibodies. (E) Correlation analyses evaluating the scoring of PD‐L1 using the 22C3 or the E1L3N antibodies in immunohistochemistry. (F) Graphs reporting the Bland‐Altman analysis evaluating the LOAs between the 22C3 and the E1L3N antibodies used in immunohistochemistry to evaluate PD‐L1 expression. G/H Graphs (G) and Table (H) reporting the number of PD‐L1 positive cells in EOC samples stained with the 22C3 and the E1L3N antibodies and evaluated by PAT1 and PAT2, as indicated. IQR = Inter Quartile Range; ICC = Intraclass Correlation Coefficient. (H) Table reporting the number of positive cells. (I) Correlation analyses evaluating the scoring of PD‐L1 using the E1L3N antibody in immunohistochemistry (IHC) or in multiplex immunofluorescence (MIF). (L) Graph reporting the Bland‐Altman analysis evaluating the LOAs between immunohistochemistry (IHC) or in multiplex immunofluorescence (MIF) in assessing PD‐L1 expression.

**FIGURE 2:**
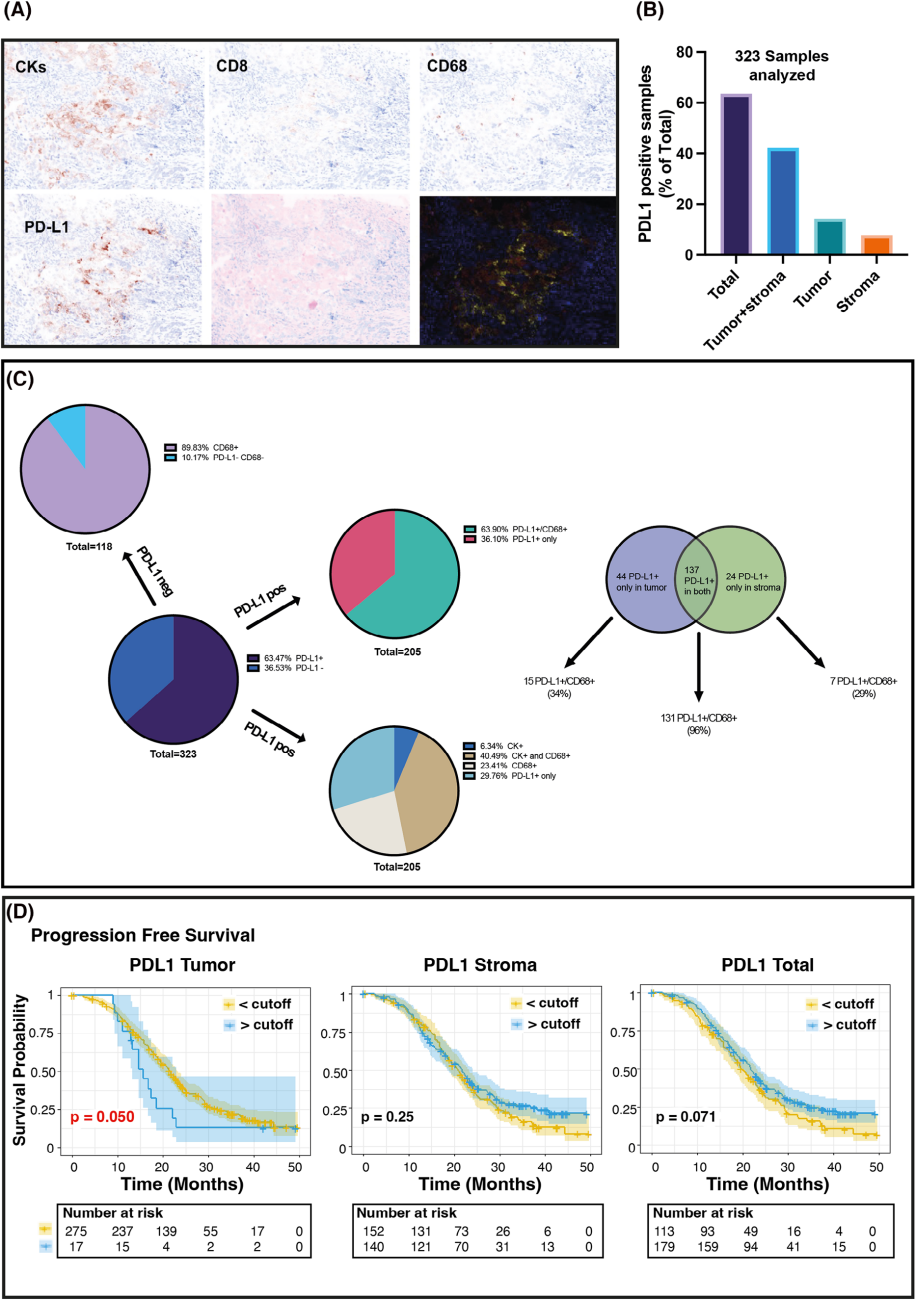
Multiplex staining evaluating tumour and stroma immune infiltration in samples from the MITO16A case material. (A) Typical image of the multiplex analyses using the OPAL Multiplex Assay coupled with multispectral image acquisition. Centralized tumour samples were processed and single FFPE tissue slides were stained for the indicated antibodies. Stained sections were acquired with a Nikon microscope coupled with a multispectral camera and acquired images were analyzed with the MANTRA software to define the number of immune cells infiltrating the tumours (PanCK‐positive areas) and the surrounding stroma. (B) Graph reporting the distribution of PD‐L1 positive cells in the analyzed samples. (C) Pie charts reporting the subtypes of PD‐L1 positive cells in the analyzed samples. (D) Kaplan Meier curves evaluating patients’ progression‐free survival based on PD‐L1 expression in the tumour (left panels), in the stroma (middle panels) or in the whole section (right panels), using the identified best cutoff.

We then asked whether PD‐L1 expression (positive vs. negative, Figure S3) and spatial distribution could have any prognostic significance in the MITO16A population, applying univariate or multivariable analysis. In univariate analyses, PD‐L1 did not significantly predict tumour prognosis for progression‐free survival (PFS) or overall survival (OS) (Table S1). However, when the model was adjusted for the clinical variables age, ECOG performance status, residual disease, FIGO stage and tumour histology, PD‐L1 negative samples in the stroma and in both stroma and tumour were associated with a significantly worse prognosis both in PFS (Hazard Ratio [HR] = 1.57, *p* = 0.003) and in OS (HR = 1.56, *p* = 0.048) (Table 2).

**TABLE 2:** Analysis of biomarkers in continuous for progression‐free and overall survival adjusted for clinical characteristics.

	Progression‐free survival			Overall survival		
MODEL PD‐L1	**HR**	**(95% CI)**	***p***	**HR**	**(95% CI)**	***p***
Tumour—Continuous linear	1.00	(0.99–1.01)	0.167	1.57	(0.65–3.76)	0.316
Tumour—Zero value	1.08	(0.79–1.49)	0.629	0.83	(0.31–2.21)	0.709
Stroma—Continuous linear	1.00	(0.99–1.01)	0.453	1.00	(0.99–1.01)	0.278
Stroma—Zero value	**1.40**	**(1.01–1.93)**	**0.044**	1.52	(0.96–2.4)	0.077
Sum—Continuous linear	1.00	(0.99–1.01)	0.109	1.00	(0.99–1.01)	0.492
Sum—Zero value	**1.57**	**(1.17–2.11)**	**0.003**	**1.56**	**(1.00–2.42)**	**0.048**

Model adjusted for age (as category < 65 vs. ≥65), ECOG performance status (0 vs. 1–2), Residual disease (None; ≤1 cm; > 1 cm; not operated), FIGO stage (III vs. IV) and Tumor histology (High‐Grade Serous vs other).

HR = Hazard Ratio.

CI = Confidence Interval.

*p* = *p*‐value.

In bold significant differences.

We then searched for the best cutoff that minimizes the p‐value of Hazard Ratio (HR) for PD‐L1 total, tumoural, and stromal expression. Very high expression of PD‐L1 (best cutoff = 117.9) in the tumour was associated with increased HR for PFS (Figure 2D and Table S2). A high number of PD‐L1^+^ cells in the tumour (HR = 2.08 *p* = 0.014), and a low number (<0.3) of PD‐L1^+^ cells in the stroma or the whole tumour section were associated with shorter PFS (HR 0.65, *p* = 0.005 for stroma and HR 0.65, *p* = 0.003 for total PD‐L1) but not OS (Figure S4 and Table S3). However, in both cases, when adjusted for overfitting HR estimates using the bootstrap‐percentile method,^10^ the associations did not maintain statistical significance (Tables S2 and S3).

This study has some limitations that include the type and number of analyzed cells, the impossibility of comparing the results with other techniques and the percentage of staining failure.

Despite these limitations, we propose MIF as a feasible technique with potential clinical utility for the analysis of the spatial distribution of selected biomarkers. Our data suggest that the absence of PD‐L1‐positive cells in the tumour, along with the presence of PD‐L1‐positive cells in the stroma of EOC, could be associated with a better prognosis when BEV is added to the standard chemotherapy. The evaluation of PD‐L1 expression using MIF in future prospective ICI trials is warranted to properly evaluate its predictive value.

## AUTHOR CONTRIBUTIONS

Conceptualization: GB and SP; Data curation: VC, FBV, EP and RDC; Formal Analysis: PC and LA; Funding acquisition: GB and SP; Investigation: VC, FBV and EP; Methodology: DR, AS, DC, GS and ADM; Project administration: GB, SP and FP; Resources: SP, DL, SCC, GA, CDA, SC and VS; Software: GB and SP; Supervision: GB and SP; Validation; Visualization: GB and FBV; Writing—original draft: GB, FBV and SP; Writing—review & editing: GB, FBV and SP. All authors read and approved the manuscript.

## CONFLICT OF INTEREST STATEMENT

The authors declare no conflict of interest.

## FUNDING INFORMATION

This work was supported by grants from: CRO Aviano Ricerca Corrente core grant (linea 1) of Ministero della Salute (G. Baldassarre); Associazione Italiana Ricerca sul Cancro (AIRC) (IG 26253) (G. Baldassarre); Alleanza Contro il Cancro (ACC) (RCR‐2022‐23682287) (G. Baldassarre); Ministero della Salute (PNRR‐MAD‐2022‐12375663) (G. Baldassarre); Ministero Università e Ricerca (MIUR) (ARS01_00568) (G. Baldassarre); Ministero della Salute (CO‐2018‐12367051) (S. Pignata); Associazione Italiana Ricerca sul Cancro (AIRC) (IG 25932) (S. Pignata); Ricerca Corrente L3/13 of Ministero della Salute (S. Pignata); Associazione Italiana Ricerca sul Cancro (AIRC) (Italy Post‐Doc Fellowship 29639 and 31335) (F. Basso‐Valentina).

## ETHICS STATEMENT

MITO16A‐MaNGO OV‐2 is a phase IV multicenter single‐arm registered trial (www.clinicaltrials.gov number: NCT01706120). The study was approved by the Ethics Committee and all patients provided written informed consent.

## Supporting information



Supporting Information

## Data Availability

Data sharing is not applicable to this article as no datasets were generated during the current study.
