# Supplementary material for: Programmed death‐ligand 1 expression and prognostic significance in bevacizumab treated ovarian cancer patients: Results from the phase IV MITO16A/MaNGO OV‐2 translational study
Source: Clin Transl Med. 2025 Jun 22;15(6):e70373. doi: 10.1002/ctm2.70373 (PMC12183328; doi:10.1002/ctm2.70373)
Supplement: Supplementary file 1 — Supporting Information [file CTM2-15-e70373-s001.pdf]

Supplementary Information

PDL1 expression and prognostic significance in Bevacizumab treated ovarian cancer patients: Results from the phase IV MITO16A/ManGO OV-2 translational study

Francesca Basso-Valentina, Vincenzo Canzonieri, Rossella De Cecio et al.

Contents:

Supplementary Patients and Methods

Supplementary Discussion

Supplementary References

Supplementary Figure S1	Consort of the study
Supplementary Figure S2	Related to Figure 1
Supplementary Figure S3	Related to Figure 2
Supplementary Figure S4	Related to Figure 2
Supplementary Table S1	Related to Table 2
Supplementary Table S2	Related to Figure 2
Supplementary Table S3	Related to Figure 2
Supplementary Table S4	Related to Figure 2
Supplementary Table S5	Related to Figure 2

Supplementary Patients and Methods

Patients.

MITO16A-MaNGO OV-2 is a phase IV multicenter single-arm registered trial (www.clinicaltrials.gov number: NCT01706120) aiming at exploring the prognostic role of clinical and selected biological factors in EOC patients treated in first line with chemotherapy (Paclitaxel + Carboplatin x6) plus BEV (15 mg/kg) for 15 months [1]. The study was designed to have 80% power to identify a potential prognostic factor able to select a favorable subgroup with a 0.60 HR, expressed in at least 20% of the population. With an alpha error of 0.01, 280 events (either PFS or OS) were required for the final analysis, and a sample size of 400 patients was planned [1]. The study was approved by the Ethic Committee and all patients provided written informed consent.

Three hundred and ninety-eight patients were enrolled in the study and tissue samples collection was centralized at the INT G. Pascale of Naples that supervised the quality controls and performed tissue processing nucleic acid extraction then providing to final investigators the biological material as necessary and described in [2, 3].

Multiplex Immunofluorescence (MIF) analyses.

Histological sections (5µm) were made from the paraffin blocks. Whole tissue sections were used to evaluate the expression on the same slide of CD8 (T Lymphocytes), CD68 (Macrophages), CD274 (PD-L1 Positive cells), and Cytokeratines (Pan-CK, tumor cells). Dapi was used to stain cell nuclei. Of the 398 patients enrolled in the MITO16A-MaNGO OV-2 phase 4 trial who agreed to donate their samples for translational studies, 42 were excluded for different reasons. Samples from 356 patients were analyzed by MIF and 64 patients were excluded at this stage for technical reasons (see Consort Figure S1).

Before proceeding with MIF, 5 µm-thick sections were deparaffinated, rehydrated and subjected to epitope retrieval through microwave treatment. To perform MIF, slides were stained with Opal 7 Immunology Discovery Kit (OP7DS1001KT, PerkinElmer) accordingly to manufacturer's instructions. Slides were subjected to MIF by consecutive staining for in the following order: CD8, CD68, PD-L1 and pan cytokeratins (PanCK). Anti CD8 and CD68 were provided in the OP7DS1001KT (PerkinElmer). Anti-PD-L1 (E1L3N®) XP® Rabbit mAb was from Cell Signalling. Anti PanCK mAb was from Roche (clone AE1/AE3/PCK26 Ventana).

Briefly, sections were rinsed, blocked with Antibody Diluent (ARD1001EA, PerkinElmer), incubated with the appropriate primary and secondary antibodies and Tyramide Signal Amplification (TSA) visualization was carried out with the marker assigned OPAL. After each staining round, antibody labelling was followed by an epitope retrieval step (using appropriate buffer, accordingly to primary antibody requirements) and a blocking step. Slides were

counterstained with Spectral DAPI reagent (PerkinElmer). MIF images were acquired using the MANTRA System (Mantra 1.0.2, PerkinElmer). Single-plex stained slides, one for each OPAL fluorochrome used in this study, and DAPI, were applied to build the spectral library. Multispectral fluorescent images were analyzed using the inForm software (inForm 2.4.1, PerkinElmer). First, MIF images were unmixed applying the spectral library and autofluorescence signal was removed. Then, images were analyzed with inForm software applying tissue segmentation, cell segmentation, and scoring of positive cells in tumor and/or stroma for each fluorochrome . At least three representative fields at 20x magnification were acquired for each sample.

Immunohistochemistry analyses.

Immunohistochemistry (IHC) was performed using 3-μm-thick histological section from 100 patients included in the MITO16A study and selected as described below in statistical analyses. Anti PD-L1 clone 22C3 (Dako) and clone E1L3N (Cell Signalling) were tested using semi-automatic and manual procedures, respectively. The antibody selection rationale aimed to compare a PD-L1 antibody suitable for mIF (E1L3N) with one already established as a companion diagnostic for immune checkpoint inhibitor (ICI) therapy in immunohistochemistry (IHC). Among the three such companion diagnostic antibodies available at the time of the study, SP263 (Ventana) was excluded because of its incompatibility with mIF protocols; 28-8 (pharmDx) and 22C3 were both compared with E1L3N. E1L3N and 22C3 provided the most reproducible and cost-effective staining results, leading to the choice of these two antibodies for further analysis.

Statistical analyses.

Sample size for methods comparison. Suggesting a moderate level of agreement between the two measurements (ICC equal to 0.70), a type 1 error alpha equal to 0.05, two methods to compare and a confidence interval for the estimated ICC from 0.6 to 0.8 sample size for antibodies comparisons was calculated to be at least equal to 101.

Statistical analyses to evaluate PD-L1 expression and prognostic significance have been performed as thoroughly described in [2]. Briefly, a histogram was used to describe the distribution. A scatterplot and a modified version of Kendall test for zero-inflated values were used to test the correlation between biomarkers [4].

Comparisons methods evaluation. The reliability of the methods (IHC and Digital) was tested by comparing clone E1L3N and clone 22C3 measured between methods and centers. Data are presented as mean ± standard deviation, median and IQR. Differences in measurement between methods were studied using Bland-Altman analysis and it were graphically summarized in terms of the Limits of Agreement (LOAs). A further indicator of agreement of the methods was determined

by the Intraclass Correlation Coefficient (ICC). ICC estimates and their 95% confident intervals were calculated on a mean-rating (k=2), absolute-agreement, 2-way mixed-effects model [5].

Analysis of prognostic association. The associations between PD-L1 expression and the clinical prognostic factors were investigate using the Wilcoxon rank test for zero-inflated data (ZIW) for dichotomous variables and the Kruskal-Wallis zero inflated (ZIKW) for categorical variables, using a permutation test. The prognostic effect of each biomarker was evaluated using Progression Free Survival (PFS) and Overall survival (OS) as endpoints.

Kaplan-Meier curves were drawn for PFS and OS and compared with a two-sided log-rank test.

To test the prognostic role for PD-L1 on both PFS and OS univariate and multivariable Cox proportional models were performed.

In univariable analysis PD-L1 was tested as a categorical variable using a biologically relevant cutoff or after searching for the best cutoff value that minimizes the p-value of Hazard Ratio (HR). The best cutoff was selected by choosing the value that minimized the p-value of HR on PFS and then applied to the OS. The multivariable analysis was performed using as covariates: age (as category <65 vs ≥65), ECOG performance status (PS) (0 vs 1-2), Residual disease (None; ≤1cm; >1cm; not operated), FIGO stage (III vs IV) and Tumor histology (high-grade serous vs other). A shrinkage procedure with 95% CI was calculated with bootstrap-percentile method [6] to adjust for over fitting HRs estimates of best cutoff categories. Data were analyzed using R software version 3.6.0 (R Foundation for Statistical Computing, Vienna, Austria) and STATA/MP 14.1 (StataCorp LP, College Station, TX).

Supplementary Discussion

Here, we used combined IHC and MIF approaches to evaluate the expression of PD-L1 in EOC patients enrolled in the MITO16A/MaNGO-OV2 trial and homogenously treated with carboplatin/taxol plus bevacizumab. This specific population of EOC patients has been scarcely studied in terms of biomarker evaluation, both for limitations in the number of controlled clinical studies and for the advent of PARP inhibitors as major player in the therapy of EOC patients in first-line settings [7]. Nevertheless, it is possible that adding bevacizumab to standard chemotherapy could modify the predictive or prognostic value of already available biomarkers (e.g. immune infiltration). The MITO16A trial was specifically designed to address this clinical unmet need. Here we present some interesting observations regarding the prognostic role of PD-L1 expression and spatial distribution that might help improving the knowledge on the impact of immune regulation in the treatment and survival of EOC patients, a topic that requires further investigation. A recent press release from Merck regarding the Phase 3 KEYNOTE-B96/ENGOT-ov65 trial , which evaluates the efficacy of pembrolizumab in platinum-resistant ovarian cancer, demonstrated a clinically meaningful improvement in OS among patients with tumors expressing PD-L1, highlighting the relevance of our work to investigate the role of PD-L1 as a biomarker. To date, the evaluation of PD-L1 expression as a companion diagnostic to identify patients who might respond to immune therapies lacks technical and analytic uniformity with possible drawbacks in the definition of PD-L1 potential predictive and prognostic value. This is particularly true in EOC patients, that in large part did not respond to immunotherapy and for whom PD-L1 has shown no predictive potential [8–14]. For instance, in patients treated with atezolizumab, the PD-L1–positive status defined as tumor-infiltrating immune cell (IC) PD-L1 expression on ≥1% of tumor area using the Ventana SP142 immunohistochemistry assay, varied from 30 to 60% in the studied populations [9–11]. Moreover, since only a very small fraction of EOC had an IC value higher than 5%, a meaningful assessment of different cutoffs for PD-L1 positivity was not possible [11]. In trials testing pembrolizumab, PD-L1 positivity was calculated using the 22C3 pharmDx antibody in IHC and applying the Combined Positive Score (CPS), defined as the number of tumor and immune PD-L1-positive cells divided by the total number of tumor cells. A CPS >1 was scored in up to 84% of analyzed cases [13, 14]. Nevertheless, neither the CPS >1 nor other ways to elaborate PD-L1 positivity demonstrated a clear prognostic value [13]. Finally, in a small study testing the efficacy of Nivolumab plus bevacizumab in relapsed EOC patients, PD-L1 expression assessed with the Dako anti PD-L1 28-8 and multiple modes of categorization with different cutoffs failed to identify a predictive role for PD-L1 [8].

Our results could explain these failures in defining the predictive role of PD-L1 in EOC. In fact, using a centralized biobank and blinded revision by two expert pathologists, we showed that two

different anti PD-L1 clones have different abilities in detecting PD-L1 expression, suggesting that there is room for improvement also in the pre-analytic pathways utilized to define PD-L1 expression. We are aware that antibody variability and specificity constitutes a limitation of the MIF technique. Nevertheless, we propose that a MIF approach, by precisely scoring on the same section which cells and in which microenvironment express PD-L1, might add prognostic and, possibly, predictive value to the diagnosis of EOC. This represents a clear advantage over other emerging strategies for biomarker discovery and validation such as Raman spectroscopy, which holds great promise of fast and label-free detection, but still needs optimization regarding the possibility to identify the precise localization of the molecules in the microenvironment and the analysis of FFPE samples [15, 16]. Moreover, its implementation in clinical pathology workflows is still not established. Importantly, we demonstrated that MIF is exploitable in the context of large multicenter clinical trials, opening the way to its further validation. To the best of our knowledge, MIF was never used before to evaluate the expression of PD-L1 in large clinical trials and our results support its added value.

Our data suggest that the absence of PD-L1 can be a predictor of worse prognosis in EOC patients treated with bevacizumab, and that this is mostly due to the absence of PD-L1$^+$/CD68$^+$ cells in the stroma (Table 2, S4 and S5), pointing to spatial distribution as a pivotal variable in determining the prognostic value of a proposed biomarker. These data are in accord with the original observation that the combination of nivolumab with bevacizumab had some activity in EOC platinum-sensitive patients especially when PD-L1 is negative, although the small number of treated patients did not allow to precisely evaluate PD-L1 predictive values [8]. Being aware of the limited significance of our KM curves with low number of patients-at-risk for PD-L1 positive tumors, we however highlight the relevance of the Cox proportional hazards models (Table 2) that demonstrates a significant association of PD-L1 absence and worse prognosis both for PFS and OS. Once more, this highlights the applicability of our results in the analysis of clinically meaningful patterns of PD-L1 expression in spatial context, offering a proof-of-concept for future validation studies.

Our MIF data also support the observation that high PD-L1 expression on tumor cells is present only in a small population of EOC patients. Multivariable analyses using the best cutoff method showed that very high expression of PD-L1 in the tumor was associated with worse PFS in bevacizumab-treated patients (HR 2.08), supporting the possibility that in this population adding immunotherapy to bevacizumab could represent a viable opportunity. This observation acquires some interest in light of the fact that high PD-L1 expression in the tumor is a better predictive biomarker for atezolizumab activity [9]. Accordingly, recent data from the EORTC 1508-GCG

phase II study suggest that patients with high PD-L1 and/or CD8+ TILs positive tumors are the ones who mostly benefit from the bevacizumab plus atezolizumab treatment [17].

On the other side, high expression of PD-L1 in the stroma was associated with better prognosis (HR 0.65), reinforcing the concept that spatial distribution of PD-L1 positive cells is important in defining its prognostic and, possibly, predictive value. We are aware that these associations lost their statistical significance after testing for multiple errors (Shrunken coefficients in Table S3), likely due to the high percentage of patients lost in the analyses for different reasons (see the consort of the study in Figure S1), and this represents a limitation of our study.

Another important point MIF allowed us to point out, is that the most relevant cell population expressing PD-L1 in EOC are cells of the monocyte/macrophage lineage ($CD68^{+}$). This observation is in accord with other recent smaller reports that used MIF in EOC and established that macrophages had the highest PD-L1 expression [18]. In this manuscript, the authors suggested that, given the spatial distribution of PD-L1-expressing macrophages, the interaction between macrophages and exhausted $CD8^{+}$ T-cell might be relevant in mediating immune suppression [18].

These observations advocate that advanced single-cell spatial analyses are of primary importance in identifying long responders to immune therapy and were confirmed by recent data showing that tumor-associated macrophages (TAMs) had different activity depending on PD-L1 expression and localization. $PD\text{-}L1^{+}$ TAMs are mature and immunostimulatory with a spatial preference to T cells. In contrast, $PD\text{-}L1^{-}$ TAMs are immunosuppressive and spatially co-localize with cancer cells. Therefore, a higher density of $PD\text{-}L1^{+}$ TAMs alone or $PD\text{-}L1^{+}/PD\text{-}L1^{-}$ TAMs ratio correlate with favorable clinical outcome in two independent cohorts of breast cancer patients [19].

We are aware that our study suffers from some limitations that include the loss of about 25% of enrolled patients for different reasons, the low potency of the statistical analyses, and the use of samples prospectively collected but retrospectively analyzed. We also point out that our observation could be specific for bevacizumab-treated EOC patients and not for patient treated with different regimens like the one including PARPi and this is something that needs to be examined in the future.

Supplementary References

1. Daniele G, Raspagliesi F, Scambia G, et al (2021) Bevacizumab, carboplatin, and paclitaxel in the first line treatment of advanced ovarian cancer patients: the phase IV MITO-16A/MaNGO-OV2A study. Int J Gynecol Cancer 31:875–882. https://doi.org/10.1136/ijgc-2021-002434

2. Califano D, Gallo D, Rampioni Vinciguerra GL, et al (2021) Evaluation of Angiogenesis-Related Genes as Prognostic Biomarkers of Bevacizumab Treated Ovarian Cancer Patients: Results from the Phase IV MITO16A/ManGO OV-2 Translational Study. Cancers (Basel) 13:5152. https://doi.org/10.3390/cancers13205152

3. Califano D, Russo D, Scognamiglio G, et al (2020) Ovarian Cancer Translational Activity of the Multicenter Italian Trial in Ovarian Cancer (MITO) Group: Lessons Learned in 10 Years of Experience. Cells 9:. https://doi.org/10.3390/cells9040903

4. Pimentel RS, Niewiadomska-Bugaj M, Wang J-C (2015) Association of zero-inflated continuous variables. Statistics & Probability Letters 96:61–67. https://doi.org/10.1016/j.spl.2014.09.002

5. Koo TK, Li MY (2016) A Guideline of Selecting and Reporting Intraclass Correlation Coefficients for Reliability Research. J Chiropr Med 15:155–163. https://doi.org/10.1016/j.jcm.2016.02.012

6. Holländer N, Sauerbrei W, Schumacher M (2004) Confidence intervals for the effect of a prognostic factor after selection of an “optimal” cutpoint. Stat Med 23:1701–1713. https://doi.org/10.1002/sim.1611

7. González-Martín A, Harter P, Leary A, et al (2023) Newly diagnosed and relapsed epithelial ovarian cancer: ESMO Clinical Practice Guideline for diagnosis, treatment and follow-up. Ann Oncol 34:833–848. https://doi.org/10.1016/j.annonc.2023.07.011

8. Liu JF, Herold C, Gray KP, et al (2019) Assessment of Combined Nivolumab and Bevacizumab in Relapsed Ovarian Cancer: A Phase 2 Clinical Trial. JAMA Oncol 5:1731–1738. https://doi.org/10.1001/jamaoncol.2019.3343

9. Moore KN, Bookman M, Sehouli J, et al (2021) Atezolizumab, Bevacizumab, and Chemotherapy for Newly Diagnosed Stage III or IV Ovarian Cancer: Placebo-Controlled Randomized Phase III Trial (IMagyn050/GOG 3015/ENGOT-OV39). J Clin Oncol 39:1842–1855. https://doi.org/10.1200/JCO.21.00306

10. Kurtz J-E, Pujade-Lauraine E, Oaknin A, et al (2023) Atezolizumab Combined With Bevacizumab and Platinum-Based Therapy for Platinum-Sensitive Ovarian Cancer: Placebo-Controlled Randomized Phase III ATALANTE/ENGOT-ov29 Trial. J Clin Oncol 41:4768–4778. https://doi.org/10.1200/JCO.23.00529

11. González-Martín A, Rubio MJ, Heitz F, et al (2024) Atezolizumab Combined With Platinum and Maintenance Niraparib for Recurrent Ovarian Cancer With a Platinum-Free Interval >6 Months: ENGOT-OV41/GEICO 69-O/ANITA Phase III Trial. J Clin Oncol 42:4294–4304. https://doi.org/10.1200/JCO.24.00668

12. Liu JF, Gaillard S, Wahner Hendrickson AE, et al (2024) Niraparib, Dostarlimab, and Bevacizumab as Combination Therapy in Pretreated, Advanced Platinum-Resistant Ovarian

Cancer: Findings From Cohort A of the OPAL Phase II Trial. JCO Precis Oncol 8:e2300693. https://doi.org/10.1200/PO.23.00693

13. González-Martín A, Chung HC, Saada-Bouzid E, et al (2024) Lenvatinib plus pembrolizumab for patients with previously treated advanced ovarian cancer: Results from the phase 2 multicohort LEAP-005 study. Gynecol Oncol 186:182–190. https://doi.org/10.1016/j.ygyno.2024.04.011

14. How JA, Dang M, Lee S, et al (2025) Pembrolizumab plus chemotherapy in frontline treatment of advanced ovarian cancer: Clinical and translational results from a phase 2 trial. Med 6:100494. https://doi.org/10.1016/j.medj.2024.07.022

15. Klamminger GG, Gérardy J-J, Jelke F, et al (2021) Application of Raman spectroscopy for detection of histologically distinct areas in formalin-fixed paraffin-embedded glioblastoma. Neurooncol Adv 3:vdab077. https://doi.org/10.1093/noajnl/vdab077

16. Zhou Q-Q, Guo J, Wang Z, et al (2024) Rapid visualization of PD-L1 expression level in glioblastoma immune microenvironment via machine learning cascade-based Raman histopathology. J Adv Res 65:257–271. https://doi.org/10.1016/j.jare.2023.12.002

17. Banerjee S, Ghisoni E, Wolfer A, et al (2025) Bevacizumab, atezolizumab and acetylsalicylic acid in recurrent, platinum-resistant ovarian cancer: the EORTC 1508-GCG phase II study. Clin Cancer Res. https://doi.org/10.1158/1078-0432.CCR-24-3368

18. Färkkilä A, Gulhan DC, Casado J, et al (2020) Immunogenomic profiling determines responses to combined PARP and PD-1 inhibition in ovarian cancer. Nat Commun 11:1459. https://doi.org/10.1038/s41467-020-15315-8

19. Wang L, Guo W, Guo Z, et al (2024) PD-L1-expressing tumor-associated macrophages are immunostimulatory and associate with good clinical outcome in human breast cancer. CR Med 5:. https://doi.org/10.1016/j.xcrm.2024.101420

Supplementary Figures and legends

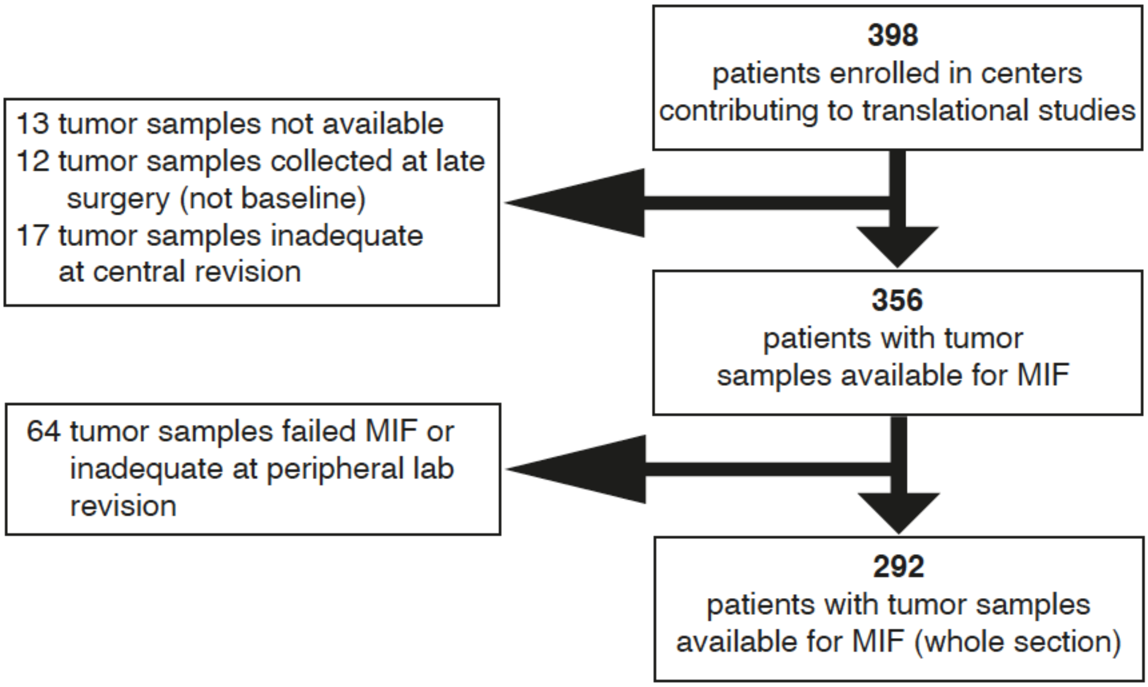

Supplementary Figure S1. Consort of the study

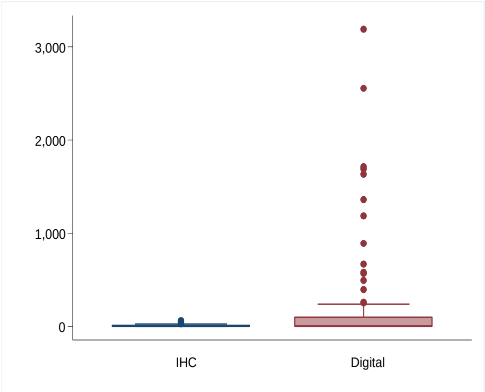

	E1L3N IHC	E1L3N Digital
Media (sd)	9,9 (14,8)	2,0 (5,3)
Median (IQR)	3,16 (0,44-11,6)	0,08 (0-0,98)
**ICC (95% CI)**	**0,45 (0,11-0,66)**	

Supplementary Figure S2.

Graph and Table reporting the number of PD-L1 positive cells in EOC samples stained with the the E1L3N antibody and evaluated by IHC and MIF, as indicated. IQR = Inter Quartile Range; ICC = Intraclass Correlation Coefficient.

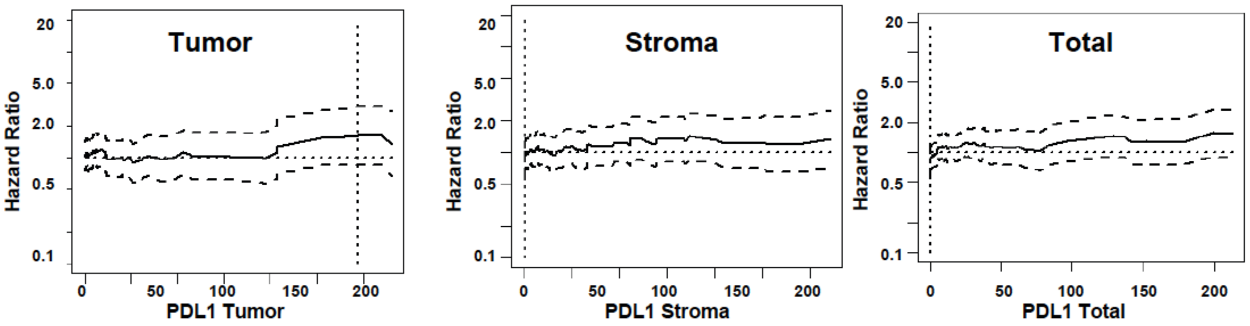

Supplementary Figure S3. Definition of best cut off for PDL1 expression and localization

Graphical representation of best cut off definition in the studied population evaluated based on the hazard ratio and the number of PDL1 positive cells in the tumor, stroma and in the whole section, as indicated.

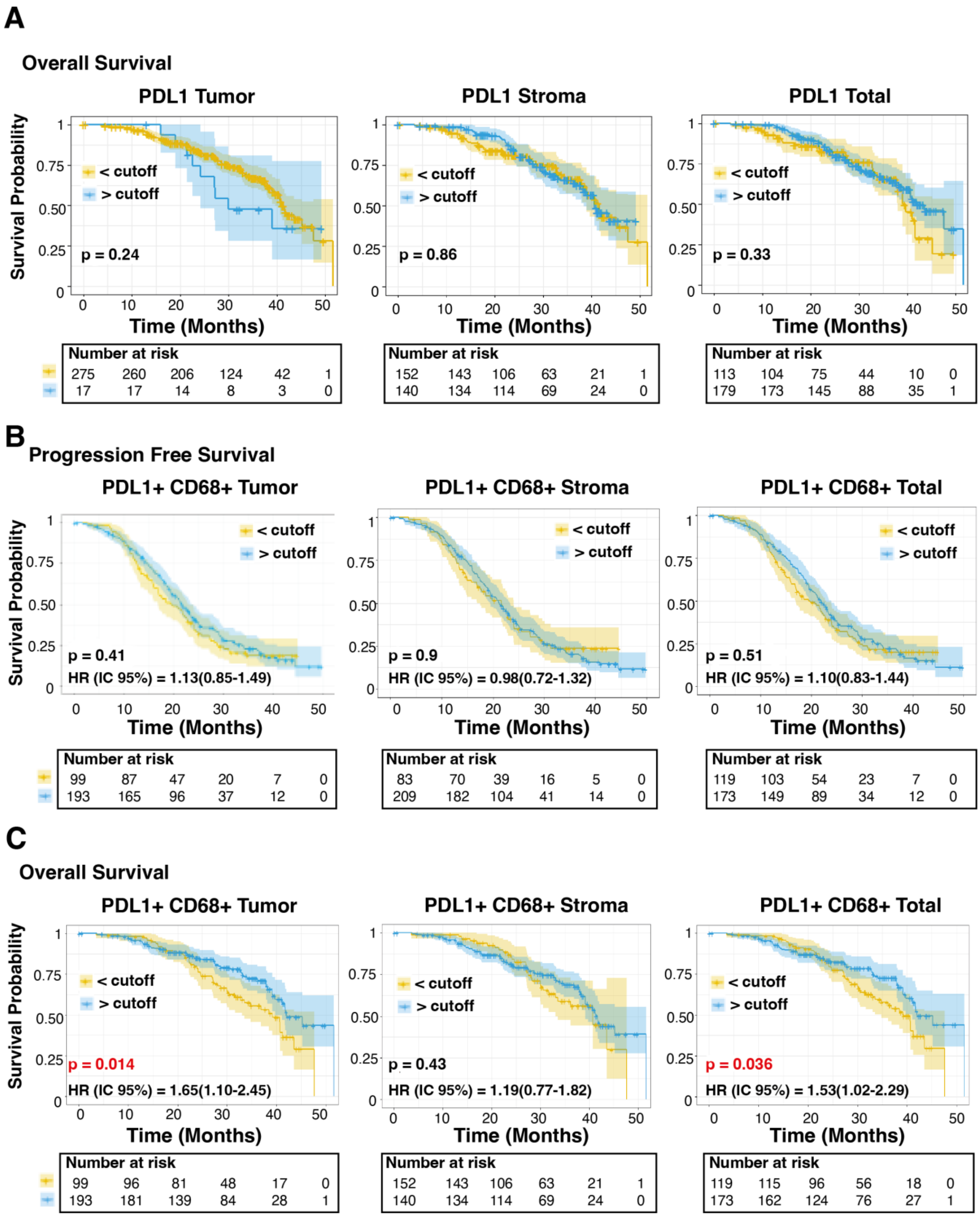

Supplementary Figure S4. Prognostic values of PD-L1 expression and CD68+PDL1+ cells number and spatial localization in analyzed cases.

A. Kaplan Meier curves evaluating patients' Overall Survival based on PD-L1 expression in the tumor (left panels), in the stroma (middle panels) or in the whole section (right panels), using the identified best cutoff.

B/C. Kaplan Meier curves evaluating patients' Progression Free Survival (PFS in **B**) and Overall Survival (OS in **C**) based on PDL1 expression in the tumor (left panels), in the stroma (middle panels) or in the whole section (right panels), using the 0 vs >0 categorization. In red are the significant differences.

Supplementary Table S1. Univariate analysis of biomarkers in continuous for Progression Free and Overall Survival.

	Progression Free Survival			Overall Survival		
**MODEL PDL1**	**HR**	**(95% CI)**	**P**	**HR**	**(95% CI)**	**P**
Tumor - Continuous linear	1.00	(0.99-1.01)	0.424	**1.01**	**(1.01-1.02)**	**0.003**
Tumor - Zero value	0.99	(0.72-1.35)	0.930	1.16	(0.71-1.88)	0.552
Stroma - Continuous linear	1.00	(0.99-1.01)	0.264	1.00	(0.99-1.01)	0.427
Stroma - Zero value	1.21	(0.89-1.66)	0.228	1.02	(0.63-1.65)	0.943
Sum - Continuous linear	1.00	(0.99-1.01)	0.100	1.00	(0.99-1.01)	0.227
Sum - Zero value	1.31	(0.98-1.74)	0.064	1.30	(8.85-1.99)	0.229

Legend to Supplementary Table S1

HR = Hazard Ratio

CI = Confidence Interval

P = p value

In bold significant differences

Supplementary Table S2: Univariate analysis of PDL1 best cut off for Progression Free Survival (PFS) and Overall Survival (OS)

**PFS**						
	**Original**			**Shrunken coefficients (bootstrap CI)**		
	**HR**	(95% CI)	P	**HR**	(95% CI)	P
**MODEL PDL1**						
**Tumor >117.9**	**1.76**	**(1.00-3.1)**	**0.050**	1.52	(0.57-4.06)	0.403
**Stroma >0.3**	0.80	(0.61-1.06)	0.128	0.88	(0.35-2.25)	0.795
**Sum >0.3**	0.78	(0.59-1.02)	0.072	0.84	(0.33-2.11)	0.712
**OS**						
**MODEL PDL1**						
**Tumor >117.9**	1.53	(0.73-3.19)	0.256	1.10	(0.15-8.31)	0.926
**Stroma >0.3**	0.75	(0.49-1.16)	0.195	0.89	(0.33-2.43)	0.820
**Sum >0.3**	0.67	(0.44-1.03)	0.069	0.76	(0.13-4.37)	0.756

Legend to Supplementary Table S2

HR = Hazard Ratio

CI = Confidence Interval

P = p value

In bold significant differences

Supplementary Table S3: Multivariable analysis of PDL1 best cut off for Progression Free Survival (PFS) and Overall Survival (OS)

**PFS**						
	**Original**			**Shrunken coefficients (bootstrap CI)**		
	**HR**	(95% CI)	P	**HR**	(95% CI)	P
**MODEL PDL1**						
**Tumor >117.9**	**2.08**	**(1.16-3.73)**	**0.014**	1.85	(0.55-6.21)	0.322
**Stroma >0.3**	**0.65**	**(0.49-0.88)**	**0.005**	0.69	(0.25-1.88)	0.469
**Sum >0.3**	**0.65**	**(0.49-0.87)**	**0.003**	0.68	(0.24-1.95)	0.476
**OS**						
**MODEL PDL1**						
**Tumor >117.9**	1.53	(0.73-3.19)	0.256	1.10	(0.15-8.31)	0.926
**Stroma >0.3**	0.75	(0.49-1.16)	0.195	0.89	(0.33-2.43)	0.820
**Sum >0.3**	0.67	(0.44-1.03)	0.069	0.76	(0.13-4.37)	0.756

Legend to Supplementary Table S3

Model adjusted for age (as category <65 vs ≥65), ECOG performance status (0 vs 1-2), Residual disease (None; ≤1cm; >1cm; not operated), FIGO stage (III vs IV) and Tumor histology (high-grade serous vs other)

HR = Hazard Ratio

CI = Confidence Interval

P = p value

In bold significant differences

Supplementary Table S4. Association of CD68 and PDL1 double positive cells with prognostic factors

	Double PDL1 CD68 Tumor			Double PDL1 CD68 Stroma			Double PDL1 CD68 Sum		
	0	>0	P	0	>0	P	0	>0	P
	N=193	N=99		N=209	N=83		N=173	N=119	
**Age Elderly**									
**<65**	138 (71.5%)	64 (64.6%)	0.23	154 (73.7%)	48 (57.8%)	**0.01**	126 (72.8%)	76 (63.9%)	0.10
**≥65**	55 (28.5%)	35 (35.4%)		55 (26.3%)	35 (42.2%)		47 (27.2%)	43 (36.1%)	
**FIGO stage**									
**III**	156 (80.8%)	78 (78.8%)	0.68	171 (81.8%)	63 (75.9%)	0.25	141 (81.5%)	93 (78.2%)	0.48
**IV**	37 (19.2%)	21 (21.2%)		38 (18.2%)	20 (24.1%)		32 (18.5%)	26 (21.8%)	
**ECOG PS**									
**0**	159 (82.4%)	75 (75.8%)	0.18	169 (80.9%)	65 (78.3%)	0.62	144 (83.2%)	90 (75.6%)	0.11
**1-2**	34 (17.6%)	24 (24.2%)		40 (19.1%)	18 (21.7%)		29 (16.8%)	29 (24.4%)	
**Tumor histology**									
**high-grade serous**	163 (84.5%)	91 (91.9%)	0.07	180 (86.1%)	74 (89.2%)	0.49	146 (84.4%)	108 (90.8%)	0.11
**other**	30 (15.5%)	8 (8.1%)		29 (13.9%)	9 (10.8%)		27 (15.6%)	11 (9.2%)	
**Residual desease**									
**None**	79 (40.9%)	36 (36.4%)	0.27	88 (42.1%)	27 (32.5%)	**0.04**	74 (42.8%)	41 (34.5%)	0.08
**<=1cm**	44 (22.8%)	16 (16.2%)		48 (23.0%)	12 (14.5%)		40 (23.1%)	20 (16.8%)	
**>1cm**	53 (27.5%)	37 (37.4%)		57 (27.3%)	33 (39.8%)		44 (25.4%)	46 (38.7%)	
**not operated**	17 (8.8%)	10 (10.1%)		16 (7.7%)	11 (13.3%)		15 (8.7%)	12 (10.1%)	

Legend to Supplementary Table S4

Age category (<65 vs ≥65)

Tumor histology (high-grade serous vs other)

FIGO stage (III vs IV)

ECOG PS (0 vs 1-2)

Residual disease (None; ≤1cm; >1cm; not operated)

For statistical analyses Wilcoxon rank test (ZIW) for zero-inflated data with permutations (except for Residual Disease where Kruskal-Wallis zero inflated (ZIKW) was applied)

In bold significant differences

Supplementary Table S5. Multivariable analysis of PDL1/CD68 in continuous for Progression Free Survival and Overall Survival.

	**Progression Free Survival**			**Overall Survival**		
	HR	(95% CI)	P	HR	(95% CI)	P
**MODEL PDL1 CD68**						
**Tumor 0**	**1**			**1**		
**Tumor >1**	0.94	(0.70-1.27)	0.704	0.90	(0.60-1.37)	0.431
**MODEL PDL1 CD68**						
**Stroma 0**	**1**			**1**		
**Stroma >1**	**0.71**	**(0.51-0.99)**	**0.043**	0.86	(0.54-1.35)	0.502
**MODEL PDL1 CD68**						
**Sum 0**	**1**			**1**		
**Sum >1**	0.86	(0.64-1.15)	0.304	1.16	(0.76-1.77)	0.481

Legend to Supplementary Table S5

Model adjusted for age (as category <65 vs ≥65), ECOG performance status (0 vs 1-2), Residual disease (None; ≤1cm; >1cm; not operated), FIGO stage (III vs IV) and Tumor histology (high-grade serous vs other)

HR = Hazard Ratio

CI = Confidence Interval

P = p value

In bold significant differences
